# Supplementary material for: Characterization of the Peer Review Network at the Center for Scientific Review, National Institutes of Health
Source: PLoS One. 2014 Aug 13;9(8):e104244. doi: 10.1371/journal.pone.0104244 (PMC4132088; doi:10.1371/journal.pone.0104244)
Supplement: Appendix S1 — List of Integrated Review Groups (IRGs) in the order given in Figure 1 . (DOCX) [file pone.0104244.s001.docx]

**Appendix S1: List of IRGs in the order given in Figure 1.**

| **IRG** | **IRG Name** |
| --- | --- |
| BCMB | Biological Chemistry and Macromolecular Biophysics |
| BST | Bioengineering Sciences and Technologies |
| CB | Cell Biology |
| GGG | Genes, Genomes and Genetics |
| IMST | Interdisciplinary Molecular Sciences and Training |
| OBT | Oncology 1 - Basic Translational |
| DKUS | Digestive, Kidney and Urological Systems |
| EMNR | Endocrinology, Metabolism, Nutrition and Reproductive Sciences |
| IDM | Infectious Diseases and Microbiology |
| IMM | Immunology |
| CVRS | Cardiovascular and Respiratory Sciences |
| MOSS | Musculoskeletal, Oral and Skin Sciences |
| OTC | Oncology 2 - Translational Clinical |
| SBIB | Surgical Sciences, Biomedical Imaging, and Bioengineering |
| VH | Vascular and Hematology |
| AARR | AIDS and Related Research |
| BBBP | Biobehavioral and Behavioral Processes |
| HDM | Healthcare Delivery and Methodologies |
| PSE | Population Sciences and Epidemiology |
| RPHB | Risk, Prevention and Health Behavior |
| BDCN | Brain Disorders and Clinical Neuroscience |
| ETTN | Emerging Technologies and Training in Neurosciences |
| IFCN | Integrative, Functional, and Cognitive Neuroscience |
| MDCN | Molecular, Cellular, and Developmental Neuroscience |
