# Supplementary material for: Characterization of the Peer Review Network at the Center for Scientific Review, National Institutes of Health
Source: PLoS One. 2014 Aug 13;9(8):e104244. doi: 10.1371/journal.pone.0104244 (PMC4132088; doi:10.1371/journal.pone.0104244)
Supplement: Appendix S2 — List of Scientific Review Groups (SRGs) in alphabetical order. (DOCX) [file pone.0104244.s002.docx]

**Appendix S2. Listing of study sections (alphabetical order).**

| **SRG** | **IRG** | **SRG Name** |
| --- | --- | --- |
| ACE | AARR | AIDS Clinical Studies and Epidemiology |
| ACTS | MOSS | Arthritis, Connective Tissue and Skin |
| ADDT | AARR | AIDS Discovery and Development of Therapeutics |
| AED | BDCN | Anterior Eye Disease |
| AICS | VH | Atherosclerosis and Inflammation of the Cardiovascular System |
| AIP | AARR | AIDS Immunology and Pathogenesis |
| AMCB | AARR | AIDS Molecular and Cellular Biology |
| ANIE | BDCN | Acute Neural Injury and Epilepsy |
| AOIC | AARR | AIDS-Associated Opportunistic Infections and Cancer |
| APDA | BBBP | Adult Psychopathology and Disorders of Aging |
| ASG | BDCN | Aging Systems and Geriatrics |
| AUD | IFCN | Auditory System |
| BACP | IDM | Bacterial Pathogenesis |
| BBM | BCMB | Biochemistry and Biophysics of Membranes |
| BCHI | HDM | Biomedical Computing and Health Informatics |
| BDMA | BST | Biodata Management and Analysis |
| BDPE | CB | Biology and Diseases of the Posterior Eye (Ended 10/2011) |
| BGES | PSE | Behavioral Genetics and Epidemiology |
| BINP | BDCN | Brain Injury and Neurovascular Pathologies |
| BMBI | BST | Biomaterials and Biointerfaces |
| BMCT | OTC | Basic Mechanisms of Cancer Therapeutics |
| BMIO | RPHB | Behavioral Medicine, Interventions and Outcomes |
| BMIT | SBIB | Biomedical Imaging Technologys |
| BMRD | HDM | Biostatistical Methods and Research Design |
| BNVT | ETTN | Bioengineering of Neuroscience, Vision and Low Vision Technologies |
| BPNS | MDCN | Biophysics of Neural Systems |
| BRLE | BBBP | Biobehavioral Regulation, Learning and Ethology |
| BSCH | AARR | Behavioral and Social Consequences of HIV/AIDS |
| BSPH | AARR | Behavioral and Social Science Approaches to Preventing HIV/AIDS |
| BTSS | SBIB | Bioengineering, Technology and Surgical Sciences |
| BVS | CB | Biology of the Visual System |
| CADO | EMNR | Cellular Aspects of Diabetes and Obesity |
| CAMP | OBT | Cancer Molecular Pathobiology |
| CASE | PSE | Cardiovascular and Sleep Epidemiology |
| CBSS | OTC | Cancer Biomarkers |
| CCHF | CVRS | Cardiac Contractility, Hypertrophy, and Failure |
| CDD | CVRS | Cardiovascular Differentiation and Development |
| CDIN | BDCN | Chronic Dysfunction and Integrative Neurodegeneration |
| CDP | OTC | Chemo/Dietary Prevention |
| CE | OBT | Cancer Etiology |
| CG | OBT | Cancer Genetics |
| CICS | CVRS | Clinical and Integrative Cardiovascular Sciences |
| CIDO | EMNR | Clinical and Integrative Diabetes and Obesity |
| CIHB | HDM | Community Influences on Health Behavior |
| CII | OTC | Cancer Immunopathology and Immunotherapy |
| CIMG | DKUS | Clinical, Integrative and Molecular Gastroenterology |
| CLHP | HDM | Community-Level Health Promotion |
| CMAD | CB | Cellular Mechanisms in Aging and Development |
| CMBG | MDCN | Cellular and Molecular Biology of Glia |
| CMBK | DKUS | Cellular and Molecular Biology of the Kidney |
| CMIA | IMM | Cellular and Molecular Immunology - A |
| CMIB | IMM | Cellular and Molecular Immunology - B |
| CMIP | SBIB | Clinical Molecular Imaging and Probe Development |
| CMIR | EMNR | Cellular, Molecular and Integrative Reproduction |
| CMND | MDCN | Cellular and Molecular Biology of Neurodegeneration |
| CNBT | BDCN | Clinical Neuroimmunology and Brain Tumors |
| CNN | BDCN | Clinical Neuroscience and Neurodegeneration |
| CNNT | BDCN | Clinical Neuroplasticity and Neurotransmitters |
| CONC | OTC | Clinical Oncology |
| CP | BBBP | Cognition and Perception |
| CPDD | BBBP | Child Psychopathology and Developmental Disabilities |
| CRFS | IDM | Clinical Research and Field Studies of Infectious Diseases |
| CSRS | CB | Cellular Signaling and Regulatory Systems |
| DBD | BDCN | Developmental Brain Disorders |
| DDR | IDM | Drug Discovery and Mechanisms of Antimicrobial Resistance |
| DEV1 | CB | Development - 1 |
| DEV2 | CB | Development - 2 |
| DIRH | HDM | Dissemination and Implementation Research in Health |
| DMP | OTC | Drug Discovery and Molecular Pharmacology |
| DPVS | BDCN | Diseases and Pathophysiology of the Visual System |
| DT | OTC | Developmental Therapeutics |
| EBIT | IMST | Enabling Bioanalytical and Imaging Technologies |
| EPIC | PSE | Epidemiology of Cancer |
| ESTA | CVRS | Electrical Signaling, Ion Transport, and Arrhythmias |
| GCAT | GGG | Genomics, Computational Biology and Technology |
| GDD | BST | Gene and Drug Delivery Systems |
| GHD | GGG | Genetics of Health and Disease |
| GMPB | DKUS | Gastrointestinal Mucosal Pathobiology |
| GVE | GGG | Genetic Variation and Evolution |
| HAI | IMM | Hypersensitivity, Autoimmune, and Immune-mediated Diseases |
| HBPP | DKUS | Hepatobiliary Pathophysiology |
| HDEP | HDM | Health Disparities and Equity Promotion |
| HIBP | IDM | Host Interactions with Bacterial Pathogens |
| HM | VH | Hypertension and Microcirculation |
| HSOD | HDM | Health Services Organization and Delivery |
| HT | VH | Hemostasis and Thrombosis |
| ICER | EMNR | Integrative and Clinical Endocrinology and Reproduction |
| ICI | CB | Intercellular Interactions |
| IHD | IMM | Immunity and Host Defense |
| III | IMM | Innate Immunity and Inflammation |
| INMP | EMNR | Integrative Nutrition and Metabolic Processes |
| IPOD | EMNR | Integrative Physiology of Obesity and Diabetes |
| IRAP | PSE | Infectious Diseases, Reproductive Health, Asthma and Pulmonary Conditions |
| ISD | BST | Instrumentation and Systems Development |
| KMBD | DKUS | Kidney Molecular Biology and Genitourinary Organ Development |
| KNOD | PSE | Kidney, Nutrition, Obesity and Diabetes |
| LAM | IFCN | Neurobiology of Learning and Memory |
| LCMI | CVRS | Lung Cellular, Molecular, and Immunobiology |
| LCOM | BBBP | Language and Communication |
| LIRR | CVRS | Lung Injury, Repair, and Remodeling |
| MABS | BST | Modeling and Analysis of Biological Systems |
| MBPP | CB | Membrane Biology and Protein Processing |
| MCE | EMNR | Molecular and Cellular Endocrinology |
| MCH | VH | Molecular and Cellular Hematology |
| MEDI | SBIB | Medical Imaging |
| MESH | BBBP | Mechanisms of Emotion, Stress and Health |
| MFSR | BBBP | Motor Function, Speech and Rehabilitation |
| MGA | GGG | Molecular Genetics A |
| MGB | GGG | Molecular Genetics B |
| MIM | CVRS | Myocardial Ischemia and Metabolism |
| MIST | CB | Molecular and Integrative Signal Transduction |
| MNG | ETTN | Molecular Neurogenetics |
| MNPS | MDCN | Molecular Neuropharmacology and Signaling |
| MONC | OBT | Molecular Oncogenesis |
| MRS | MOSS | Musculoskeletal Rehabilitation Sciences |
| MSFA | BCMB | Macromolecular Structure and Function A |
| MSFB | BCMB | Macromolecular Structure and Function B |
| MSFC | BCMB | Macromolecular Structure and Function C |
| MSFD | BCMB | Macromolecular Structure and Function D |
| MSFE | BCMB | Macromolecular Structure and Function E |
| MTE | MOSS | Musculoskeletal Tissue Engineering |
| NAED | AARR | NeuroAIDS and other End-Organ Diseases |
| NAL | IFCN | Neurotoxicology and Alcohol |
| NAME | PSE | Neurological, Aging and Musculoskeletal Epidemiology |
| NANO | BST | Nanotechnology |
| NCF | MDCN | Neurogenesis and Cell Fate |
| NCSD | CB | Nuclear and Cytoplasmic Structure/Function and Dynamics |
| NDPR | MDCN | Neurodifferentiation, Plasticity, Regeneration and Rhythmicity |
| NMB | IFCN | Neurobiology of Motivated Behavior |
| NNB | IFCN | Neuroendocrinology, Neuroimmunology and Behavior |
| NNRS | IFCN | Neuroendocrinology, Neuroimmunology, Rhythms and Sleep |
| NOIT | ETTN | Neuroscience and Ophthalmic Imaging Technologies |
| NOMD | MDCN | Neural Oxidative Metabolism and Death |
| NPAS | BDCN | Neural Basis of Psychopathology, Addictions and Sleep Disorders |
| NRCS | HDM | Nursing and Related Clinical Sciences |
| NTRC | MDCN | Neurotransporters, Receptors, and Calcium Signaling |
| ODCS | MOSS | Oral, Dental and Craniofacial Sciences |
| PBKD | DKUS | Pathobiology of Kidney Disease |
| PCMB | GGG | Prokaryotic Cell and Molecular Biology |
| PDRP | RPHB | Psychosocial Development, Risk and Prevention |
| PMDA | BDCN | Pathophysiological Basis of Mental Disorders and Addictions |
| PN | EMNR | Pregnancy and Neonatology |
| PRDP | RPHB | Psychosocial Risk and Disease Prevention |
| PTHE | IDM | Pathogenic Eukaryotes |
| RIBT | CVRS | Respiratory Integrative Biology and Translational Research |
| RPIA | RPHB | Risk, Prevention and Intervention for Addictions |
| RTB | OTC | Radiation Therapeutics and Biology |
| SAT | SBIB | Surgery, Anesthesiology and Trauma |
| SBCA | BCMB | Synthetic and Biological Chemistry A |
| SBCB | BCMB | Synthetic and Biological Chemistry B |
| SBDD | MOSS | Skeletal Biology Development and Disease |
| SBSR | MOSS | Skeletal Biology Structure and Regeneration |
| SCS | IFCN | Somatosensory and Chemosensory Systems |
| SEIR | PSE | Societal and Ethical Issues in Research |
| SMEP | MOSS | Skeletal Muscle and Exercise Physiology |
| SMI | IFCN | Sensorimotor Integration |
| SPC | IFCN | Mechanisms of Sensory, Perceptual, and Cognitive Processes |
| SPIP | RPHB | Social Psychology, Personality and Interpersonal Processes |
| SSPS | PSE | Social Sciences and Population Studies |
| SYN | MDCN | Synapses, Cytoskeleton and Trafficking |
| TAG | GGG | Therapeutic Approaches to Genetic Diseases |
| TCB | OBT | Tumor Cell Biology |
| TME | OBT | Tumor Microenvironment |
| TPM | OBT | Tumor Progression and Metastasis |
| TTT | IMM | Transplantation, Tolerance, and Tumor Immunology |
| UGPP | DKUS | Urologic and Genitourinary Physiology and Pathology |
| UKGD | DKUS | Urologic and Kidney Development and Genitourinary Disease |
| VACC | AARR | HIV/AIDS Vaccines |
| VB | IDM | Vector Biology |
| VCMB | VH | Vascular Cell and Molecular Biology |
| VIRA | IDM | Virology - A |
| VIRB | IDM | Virology - B |
| VMD | IMM | Vaccines Against Microbial Diseases |
| XNDA | DKUS | Xenobiotic and Nutrient Disposition and Action |
